# *Bulleidia extructa* Periprosthetic Hip Joint Infection, United States

**DOI:** 10.3201/eid1907.130078

**Published:** 2013-07

**Authors:** Benjamin Kloesel, Margaret Beliveau, Robin Patel, Robert T. Trousdale, Irene G. Sia

**Affiliations:** Mayo Clinic, Rochester, MN, USA

**Keywords:** Periprosthetic joint infection, Bulleidia extructa, total hip arthroplasty, anaerobic bacteria, infection, United States, bacteria

**To the Editor:** *Bulleidia extructa* is an obligately anaerobic, nonmotile, non–spore-forming gram-positive bacillus first described in 2000 by Downes et al. (*1*), after having isolated a bacterium from the oral cavity of persons with periodontitis and dentoalveolar abscesses that did not correspond to any known species. After phenotypic and genetic characterization, the investigators proposed a new genus, *Bulleidia*, and the species *B. extructa*. Since then, additional reports have associated the organism with oral infections, specifically periodontal disease (*2*–*5*). While *B. extructa*’s association with human periodontal disease is well documented, the bacterium has so far not been implicated in other pathogenic processes. We report here a case of a total hip arthroplasty infection caused by *B. extructa* in an immunocompetent patient.

In November 2010, an 82-year-old man with a non-cemented right total hip arthroplasty that was performed 26 years previously was evaluated for right hip pain. He had been in his usual state of health without any complaints until a month earlier, when he lost his footing and hyperabducted his hip joints, involuntarily performing a split, while washing a boat cover with a power washer. Since then, he reported right hip pain that somewhat limited his mobility.

Physical examination revealed an antalgic gait, mild swelling of the right lower extremity, and impaired hip mobility related to pain on the right side, specifically with extension, flexion, abduction, and adduction. Results of the patient’s blood work were notable for normocytic anemia (hemoglobin 10.6 g/dL), thrombocytosis (459 × 10^9^/L), elevated erythrocyte sedimentation rate (101 mm/h), and elevated C-reactive protein (88.7 mg/L). Leukocyte count was within normal limits (9.6 × 10^9^ cells/L). An ultrasound examination of the right hip joint showed extensive synovitis and a large, 4.3 × 5.0 × 5.1–cm vascular mass extending anteriorly from the joint space. Aspiration of the joint space yielded 1 mL of blood-stained fluid with 111,595 cells/µL (95% neutrophils, 5% monocytes/ macrophages). Anaerobic bacterial culture grew a gram-positive bacillus identified as *B. extructa* by partial 16S rRNA sequencing. DNA was prepared for PCR amplification by using PrepMan Ultra (Applied Biosystems, Foster City, CA, USA) and amplified and bidirectionally sequenced by using primers 5′-TGGAGAGTTTGATCCTGGCTCAG-3′ and 5′-TACCGCGGCTGCTGGCAC-3′. The generated 484-bp sequence differed by 2 bp from 483 bp of available sequence from *B. extructa* GenBank accession no. AF220064. The isolate was susceptible to penicillin, clindamycin, and metronidazole by using E-test.

The patient underwent total hip arthroplasty resection. Intraoperatively, purulence was noted upon entering the hip joint. Histopathologic examination of removed tissue revealed acute inflammation. Five hip tissue specimens were obtained for culture; 3 specimens yielded *B. extructa*. Six weeks of intravenous ceftriaxone treatment was prescribed, and the patient was instructed to revisit a dentist for a full dental examination. Before seeking treatment for this episode, he reported that he was seeing a dentist on a regular basis and denied any recent dental surgery or infections.

The patient was seen in a follow-up visit 2 months after reimplantation surgery; at that time, he reported minimal pain and had begun to bear weight on the affected side. There was no evidence for infection recurrence.

Periprosthetic joint infections are a major complication after joint replacement. The number of procedures for total hip and knee replacements has increased during the past 13 years (*6*). This trend is accompanied by an increase in the total number of periprosthetic joint infections, even though the overall percentage of this complication is low (*7*). The most commonly isolated organisms in periprosthetic joint infections are gram-positive cocci, specifically *Staphylococcus aureus* and *S.* *epidermidis* (*8*). In a retrospective review, Moran et al. (*9*) examined the microbiological spectrum of 112 patients undergoing debridement and irrigation for a periprosthetic joint infection (hip [52], knee [51], elbow [4], ankle [3], shoulder [2]) at a tertiary care center in the United Kingdom during 1998–2003. The most frequently isolated microorganisms were coagulase-negative staphylococci (47%) followed by methicillin-sensitive *S. aureus* (44%), methicillin-resistant *S. aureus* (8%), aerobic gram-negative organisms (8%), and anaerobes (7%). Thirty-seven percent of patient specimens grew multiple microorganisms.

We document the ability of *B. extructa* to cause an infection beyond its usual habitat, the oral flora. We hypothesize that the infection in this patient might have developed from hematogenous seeding in which an undiscovered and asymptomatic oral infectious nidus might have served as the seeding focus while mild trauma to the hip could have facilitated access to the joint space.
